# System Effects of Mental Health Agency Expenditures and Mental Health Parity Legislation at the State Level

**DOI:** 10.1007/s11414-025-09949-z

**Published:** 2025-05-27

**Authors:** Jenna Morales Ledbetter, Ronald W. Manderscheid

**Affiliations:** 1 Bloomberg School of Public Health, Johns Hopkins University, Baltimore, MD USA; 2 Suzanne Dworak-Peck School of Social Work, University of Southern California, Los Angeles, CA USA

**Keywords:** Parity, Mental health, Access to care

## Abstract

The escalating mental health crisis in the USA has left over fifty percent of adults with a mental illness without mental health services. Federal mental health parity legislation addresses financial barriers to mental healthcare by requiring that insurance coverage for mental health services is equivalent to coverage for other medical services. Using data from the top ten and bottom ten states ranked by per capita State Mental Health Agency expenditures, this paper examines the impact of parity implementation and enforcement on three system-level access to care measures: (1) mental health workforce availability, (2) percent of state population living in a mental health shortage area, and (3) percent of total health expenditure spent on mental health by state agencies. As hypothesized, the top ten states had more comprehensive parity implementation and enforcement and a larger allocation of total health expenditures to mental health (*p* = 0.0002). The other two measures did not show a significant difference but trended in the direction of greater workforce availability (*p* = 0.11) and fewer residents living in mental health provider shortage areas (*p* = 0.054) among the top ten states compared to the bottom ten states. Using the scope of mental health parity alone, all three access-to-care measures were significantly better among states with comprehensive parity compared to states without comprehensive parity. These findings highlight the critical roles of financial investment, policy prioritization, and enhanced mental health infrastructure in addressing access to mental healthcare.

## Introduction

In 2021, a staggering one-fifth of adults in the USA were living with a mental illness.^1^ Among these 57.8 million individuals, fewer than one-half of them received mental health services.^1^ The urgency of addressing mental health in the USA has grown, particularly as rates of anxiety and depression symptoms, drug overdoses, and alcohol-induced deaths surged during and after the COVID-19 pandemic.^2^ Of particular concern is the stark increase in death by suicide, which has risen by 35.2% over the past two decades, reaching a record high in 2022.^3^

The lack of access to adequate mental health services for millions of Americans exacerbates the escalating prevalence of mental health disorders. Mental health parity regulations are actively addressing the issue of access to care and aim to ensure equitable coverage for individuals with mental health disorders. The Mental Health Parity and Addiction Equity Act (MHPAEA) seeks to eliminate discriminatory practices by insurance providers by requiring that plans offering behavioral health benefits do so with the same level of coverage as physical health treatment.^4^

This work explores the impact of State Mental Health Agency expenditures and state-level mental health parity implementation on key health system measures. This report provides valuable insight to inform future mental health research and policy at both the state and federal levels.

### Background

As the prevalence of mental health disorders grows, an increasing number of individuals face significant barriers to accessing the care they need, leaving many without access to care at all.^5^ Addressing these barriers, particularly financial ones, is critical to ensure that individuals with mental health and substance use disorders receive the necessary care. Federal parity laws have been enacted to address these financial obstacles by mandating that insurance plans offering mental health or substance use disorder benefits do so on par with coverage for physical health.^4^

The Mental Health Parity Act of 1996 (MHPA) marked the first federal legislation addressing parity in mental health coverage. Despite its significance, the MHPA was limited in scope as it applied exclusively to large group plans, addressed only financial limits, and lacked an explicit requirement of parity for substance use disorder treatment.^4^

A significant parity advancement came with the 2008 passage of the Mental Health Parity and Addiction Equity Act (MHPAEA). This legislation expanded existing parity regulations to include substance use disorders and addressed both quantitative and nonquantitative treatment limits, thereby promoting more equitable access to care.^6,7,4^

In 2010, the Affordable Care Act (ACA) further expanded federal parity by including individual and small-group plans and designating mental health and substance use disorder services as “essential health benefits,” thereby ensuring their coverage under qualifying plans.^8^ Despite this progress, to date, individuals covered under Medicaid, Medicare, self-funded, grandfathered, and exempt small group plans remain outside the scope of federal parity.^4^

While federal parity laws apply nationwide, the implementation and enforcement of parity vary from state to state. In some states, mental health and substance use disorder coverage is mandated, while in others, these services are optional.^9^ Additionally, some states limit the scope of parity by mandating coverage for only a specified list of mental illnesses or limiting which mental health and substance use disorder services fall under parity.^9,10^ Conversely, other states have expanded parity, making it more comprehensive than federal requirements.^10,4^ Due to the varied implementation of parity across states, it is essential to analyze mental health parity at the state level to understand how these differences impact system quality indicators.

This paper will examine State Mental Health Agency (SMHA) expenditures as a predictor variable for parity implementation and enforcement, as well as system-level outcomes. SMHAs are responsible for organizing and administering mental health services for individuals with mental illnesses and substance use disorders.^11^ These expenditures vary significantly by state, with expenditures highest at $6,183 per individual served in the Northeast and lowest at $2,898 per individual served in the South.^12^ For this analysis, SMHA expenditures will serve as an indicator of state mental health infrastructure and as a basis for ranking states.

State mental health agency (SMHA) expenditures reflect a complex interplay of factors, including states’ broader orientation toward mental health, available financial resources, staffing capacities, and existing policy frameworks. These agencies are the primary public funders of mental health services, and their spending decisions are shaped by legislative priorities, administrative structures, prevailing social attitudes toward mental illness, and the role of public health systems.^13,14^ Differences in expenditures can also stem from whether a state emphasizes institutional care versus community-based services, the degree of Medicaid expansion, and workforce availability.^15,16^ As such, SMHA spending is both a reflection of and a mechanism for advancing mental health priorities within a state.

### Study aim

This paper examines the impact of mental health parity on the effectiveness of state mental health systems. Specifically, it will examine how expenditures by State Mental Health Agencies impact the implementation and enforcement of state-level parity and how these factors collectively impact key health system measures. The health system measures being considered are mental health workforce availability, the percentage of the state population residing in mental health provider shortage areas, and the proportion of total health expenditures allocated to SMHAs. This is the first investigation to extend the study of state-level parity implementation to specific system-level impacts.

### Hypotheses

The following hypotheses guided this research:

- States with higher State Mental Health Agency (SMHA) expenditures will have more comprehensive mental health parity implementation and enforcement.
- States with greater SMHA expenditures will have more comprehensive mental health parity and better functioning mental health systems as indicated by increased mental health workforce availability, a lower percentage of the state population living in a mental health provider shortage area, and a higher percentage of total state health expenditures allocated to mental health.
- States with comprehensive mental health parity will have better functioning mental health systems, as indicated by increased mental health workforce availability, a lower percentage of the state population living in a mental health provider shortage area, and a higher percentage of total state health expenditures allocated to mental health.

## Methods

### Ethics approval

Clinical trial number: not applicable

### Study design

The research aims to evaluate how SMHA expenditures influence the implementation of state-level mental health parity legislation and system-level access to care measures. To achieve this, the authors ranked states based on their per capita SMHA expenditures, identifying the top ten and bottom ten states for mental healthcare infrastructure. They then analyzed the scope and effectiveness of parity implementation in each state in terms of legislation, regulation, and enforcement. To identify the impact of greater per capita expenditure and more extensive parity, authors looked at several system-level access to care measures: (1) mental health workforce availability, (2) the percentage of the state population living in a mental health professional shortage area, and (3) the percentage of total health expenditures allocated to SMHAs. Additionally, they conducted case studies on selected states from both the top and bottom rankings to provide deeper insights into the factors affecting mental healthcare access.

Specifics on the variables and data sources are reported below.

### State rankings

States were ranked according to per capita expenditures by SMHAs, using data from the fiscal year 2019.^12^ This metric served as an indicator of each state’s mental health infrastructure. Washington, DC, was excluded from the ranking due to incomplete data across outcome measures. Of note, Arizona’s mental health expenditure includes substance use disorder expenditure. Expenditures in New York, Pennsylvania, Maine, Iowa, Maryland, and Ohio include funds for mental health services for incarcerated individuals. Montana and Ohio expenditures do not include Medicaid Revenues for Community Programs. Montana and Nevada expenditures do not include children’s expenditures.^12^

### Parity implementation

To assess parity implementation and enforcement across states, the authors utilized The Kennedy Forum’s Parity Track database. States were evaluated based on the comprehensiveness of their legislation, regulation, and enforcement practices. Comprehensive legislation was designated “yes,” while limited legislation was designated as such. A designation of limited legislation was used when parity only applied to plans already providing behavioral health coverage, when parity did not expand beyond government employees, when only specific mental health conditions were covered, or when financial requirements were present that were not present for other medical services. The Kennedy Forum’s Parity Track database monitors whether states have published enforcement actions concerning mental health parity legislation.^10^ For states that did not have published enforcement actions according to the Parity Track database, authors conducted an independent search to ensure the accuracy of the information in the database. No further enforcement actions were identified in any of these states.

### System effects

#### Mental health workforce availability

Mental health workforce availability measures the population per one provider. Providers in this measure include “psychiatrists, psychologists, licensed clinical social workers, counselors, marriage and family therapists, and advanced practice nurses specializing in mental healthcare”.^17^ This data, sourced from the 2021 National Provider Identification Survey, solely considers the presence of providers and does not consider access measures, including accepting new patients, waitlists, or insurance coverage.

#### Percent of state population living in a mental health professional shortage area

Data on the percentage of the state population living in a mental health professional shortage area were obtained from the Bureau of Health Workforce of the Health Resources and Services Administration (HRSA).^18^ For mental health, the population-to-provider ratio to be considered a HPSA is 30,000 to 1. Most HPSA designations specific to mental health consider only psychiatrists and do not consider other types of providers.^19^ The population of the designated HPSAs for mental healthcare was calculated by the Bureau of Health Workforce, and a percentage of the total state population was calculated. This data was last updated in 2023.

#### Percent of total health expenditure spent on mental health by state agencies

This metric was calculated using data from the NRI report on State Mental Health Agency Revenues and Expenditures ^12^ and the Centers for Medicare & Medicaid Services (CMS) National Health Expenditure Data.^20^ The denominator in this calculation is total per capita health expenditure, which came from CMS and included both private and publicly funded individual healthcare services.^21^ The numerator is per capita expenditure by State Mental Health Agencies, which came from the NRI report.^12^ The authors ran this calculation using 2019 data for both total expenditure and mental health expenditure.

### Case studies

In-depth case studies on the landscape of mental healthcare were conducted for Vermont, Arizona, Colorado, and Idaho (see Appendix). The authors selected two states to explore among the top ten and two among the bottom ten. Among the top ten states, Vermont was chosen as it was the state with the highest SMHA expenditure that also had evidence of comprehensive mental health parity legislation, regulations, and enforcement.^10^ Arizona was chosen because despite being among the top ten states for SMHA expenditure, legislation was limited, there was no published enforcement, and parity was only required by plans that already cover behavioral health services.^10^ Among the bottom states, Colorado was chosen because individual plans, small employer, and large employer fully insured plans must adhere to parity legislation, making it one of the more extensive examples of parity implementation among the bottom states.^10^ Idaho was chosen for a case study due to the incredibly limited nature of parity. In Idaho, only plans for state government employees and their nuclear families require parity.^10^

### Analysis

States were ranked according to SMHA expenditures, and the top ten and bottom ten states were used in the analysis. The authors compared parity implementation and enforcement between the top and bottom states, focusing on the proportion of states with limited versus comprehensive parity legislation and the presence or absence of parity enforcement.

The authors first analyzed the relationship between SMHA expenditures and parity to determine whether states with higher expenditures also demonstrated greater implementation of parity. The findings indicated that the relationship between expenditures and parity was not random. Based on this, the authors examined how SMHA expenditure rankings aligned with outcome measures. They then extended their analysis by assessing the parity measures directly.

To analyze the system quality indicators, the authors conducted Mann–Whitney *U* tests to compare indicators between the top ten and bottom ten states ranked by per capita SMHA expenditures. Mann–Whitney *U* tests were used because the test does not make potentially unfounded assumptions about the underlying distribution of the data.

To separate the impact of parity legislation from SMHA expenditures, the authors ran a second set of Mann–Whitney *U* tests to compare the system quality indicators between states with and without comprehensive parity.

All analyses used a significance level of 0.05. The findings from the case studies supplemented and provided more detail on the quantitative findings from this study (see Appendix).

## Results

### State rankings

All fifty states were ranked using per capita expenditures by State Mental Health Agencies (SMHAs), and the top ten and bottom ten states were used in the analysis. The ten states with the largest per capita expenditures were Alaska, Vermont, New York, Pennsylvania, Arizona, Oregon, Maine, Iowa, Montana, and Maryland, with per capita expenditures ranging from $462.46 in Alaska to $247.86 in Maryland. Conversely, the ten states with the smallest per capita expenditures were Kentucky, Ohio, Colorado, Illinois, Texas, Nevada, Louisiana, Arkansas, Florida, and Idaho, with per capita expenditures ranging from $47.95 in Kentucky down to $38.00 in Idaho (see Table 1).^12^

**Table 1:** Separate rankings for the top ten states and bottom ten states based upon per capita expenditures for behavioral health

State	Ranking	Per capita mental health expenditure by state agency^1^
***Top 10***		
Alaska	1	462.46
Vermont	2	411.12
New York	3	353.40
Pennsylvania	4	341.81
Arizona	5	337.13
Oregon	6	293.99
Maine	7	278.81
Iowa	8	269.53
Montana	9	265.93
Maryland	10	247.86
Top 10 Average	-	326.20
***Bottom 10***		
Kentucky	41	47.95
Ohio	42	45.44
Colorado	43	45.30
Illinois	44	44.93
Texas	45	41.74
Nevada	46	41.39
Louisiana	47	41.23
Arkansas	48	41.04
Florida	49	39.86
Idaho	50	38.00
Bottom 10 Average	-	42.69
Difference	-	283.52 (p < 0.0003)

^1^NRI (2022). FY 2019 State Mental Health Agency Revenues and Expenditures. NRI’s 2020–2021 State MH Profile Highlights

### Parity implementation

The first hypothesis stated that states with higher SMHA expenditures would have more comprehensive parity implementation. The results supported this hypothesis. Greater mental health expenditure was correlated with more comprehensive parity in terms of legislation, regulation, and enforcement (see Table 2). Among the top ten states, only three states (Arizona, Iowa, and Montana) had limited parity legislation. In contrast, all ten of the bottom states (Kentucky, Ohio, Colorado, Illinois, Texas, Nevada, Louisiana, Arkansas, Florida, and Idaho) had limited parity legislation. Furthermore, four of the top ten states (Vermont, New York, Pennsylvania, and Oregon) had published data on parity enforcement, while only one of the bottom states (Illinois) had data on parity enforcement.^10^ These findings support the hypothesis that, in the authors’ estimation, states with greater mental health expenditures have more comprehensive parity implementation in terms of both legislation and enforcement.

**Table 2:** State parity legislation, regulation, and enforcement for the top ten states and bottom ten states^1^

State	Legislation?	Regulations?	Enforcement?	Scope of parity
***Top 10***				
Alaska	Yes	Yes	Not published	Small and large employer fully insured plans must have behavioral health coverage at the level required by Federal Parity. Small and large employer fully insured plans must offer optional SUD coverage
Vermont	Yes	Yes	Yes	Individual plans, small and large employer fully insured plans, and state plans are required to cover all behavioral health conditions included in the ICD at the level required by Federal Parity. Behavioral health deductible cannot be different than other medical care
New York	Yes	Yes	Yes	Mental health disorders must be covered by small and large employer fully insured plans equivalently to other health conditions. There are also specific coverage requirements for inpatient and outpatient visits. Individual plans and small and large employer plans must cover SUD services without treatment limitations or financial requirements more burdensome than other medical care
Pennsylvania	Yes	Yes	Yes	Plans must meet specific visit coverage requirements for serious mental illness and the annual and lifetime maximum of coverage cannot differ from other medical care
Arizona	Limited	Yes	Not published	Plans are not required to cover behavioral health services but plans that do cannot have annual or lifetime maximums that are more burdensome than those for other medical care. This does not apply to substance use disorder services and only applies to large employer fully insured plans
Oregon	Yes	Yes	Yes	Large and small employer fully insured plans are required to cover behavioral health no more strictly than other medical conditions. Plans are also required to cover out-of-network providers, regardless of if in-network providers are providing the same services, though they don't need to be covered at the same rate as in-network services
Maine	Yes	Yes	Not published	Small and large employer fully insured plans are required to cover medically necessary behavioral health services (this includes mental health services and SUD services). Must be covered equivalently to other medical conditions. Individual plans must offer optional mental health coverage for specific conditions
Iowa	Limited	Yes	Not published	Large employer fully insured plans are required to cover services for specific severe mental disorders. Small employer plans can optionally provide coverage for mental illness, but if they do, the coverage must be at a level equivalent to physical health coverage. Substance use disorders and all mental health disorders are required to be covered for veterans
Montana	Limited	Yes	Not published	Large and small employer plans and individual plans must cover specific severe mental illnesses at an equivalent level as physical health disorders. Other mental health conditions must be covered with specific limitations for large and small fully insured plans
Maryland	Yes	Yes	Not published	Small and large employer fully insured plans and individual plans must provide coverage for behavioral health services and they must be covered at the same level as services for other health conditions. Managed care cannot be used for behavioral health conditions if it is not used for physical health conditions
***Bottom 10***				
Kentucky	Limited	Yes	Not published	Large and small employer fully insured plans and individual plans are required to offer mental health coverage, and if coverage is chosen, it must be covered at the same rate as physical health services. Small employer fully insured plans also must offer optional treatment for alcoholism, but not for other SUD
Ohio	Limited	Yes	Not published	Individual plans, small and large employer fully insured plans, and self-insured plans must cover a specific list of mental health disorders, and they must be covered no less extensively than physical health conditions
Colorado	Limited	Yes	Not published	Individual plans, small and large employer fully insured plans, and self-insured plans must cover a specific list of mental health disorders, and they must be covered no less extensively than physical health conditions
Illinois	Limited	Yes	Yes	Individual plans and large and small employer fully insured plans that provide behavioral health coverage cannot employ restrictions for these services that are stricter than for physical health services. Large employer plans have mandates for coverage for SUD and a list of serious mental illnesses when there is a medical necessity
Texas	Limited	Yes	Not published	Parity in mental health coverage is required for large and small employer fully insured plans but only for specific conditions. SUD treatment must be covered at the same level as other medical care, but for a given individual treatment is only required to be covered three times during the lifetime
Nevada	Limited	Yes	Not published	Individual and small employer fully insured plans must cover specific mental health conditions. No specifications on what this coverage must include. Small employer fully insured plans must cover SUD services, individual plans must offer it
Louisiana	Limited	Yes	Not published	Self-insured plans and large and small employer fully insured plans must cover specific mental health conditions in at least an equivalent manner to other medical conditions. Only large employer plans have regulations regarding annual and lifetime maximums. SUD coverage must be optional for large and small employer fully insured plans
Arkansas	Limited	Yes	Not published	Individual plans and small employer plans are required to offer optional coverage for behavioral health with no greater financial requirements or annual or lifetime maximums than other medical care. Reimbursement rates for mental health providers are not required to be equivalent to those for other medical services. Specific utilization requirements can be used even if not used for other medical conditions. Only small employer fully funded insurance plans are required to offer SUD services
Florida	Limited	Yes	Not published	Small and large employer fully funded plans are required to offer optional coverage for mental health treatment with specific visit limitations and annual financial maximums. Small and large plans must offer optional SUD treatment but there are both financial and visit limitations
Idaho	Limited	Yes	Not published	Only plans for state government employees and their spouses and children require coverage in an equivalent way to other medical conditions. This coverage applies only to a small list of serious mental disorders
^1^The Kennedy Forum. *State Parity Reports*. Parity Track				

### Systems of care

The second hypothesis was that states with greater expenditures by SMHAs and more comprehensive parity would have better functioning mental health systems. This was measured through three system quality indicators: (1) mental health workforce availability, (2) percent of the state population living in a mental health provider shortage area, and (3) percent of total health expenditure spent on mental health by SMHAs (see Table 3). For mental health workforce availability, the average population per provider in the top ten states was 330, compared to 424 in the bottom ten states, showing a difference of 94 individuals per provider (*p* = 0.11). Although this difference is notable, it was not statistically significant.

**Table 3:** Mental health workforce availability, percent of state population living in a mental health provider shortage area, and percent of health expenditure spent on behavioral health for the top ten states and bottom ten states

State	Mental health workforce availability (population per 1 provider)^1^	% of state population living in mental health provider shortage areas^2^	% of total health expenditure spent on mental health by state agencies^3,4^
***Top 10***			
Alaska	160	51.66	3.40
Vermont	200	0	3.38
New York	310	21.22	2.73
Pennsylvania	410	13.39	3.06
Arizona	660	38.59	4.13
Oregon	170	39.03	3.05
Maine	190	19.32	2.42
Iowa	570	57.57	2.93
Montana	300	53.10	2.70
Maryland	330	21.09	2.40
**Top 10 Avg**	***330***	***31.50***	***3.02***
***Bottom 10***			
Kentucky	390	74.29	0.49
Ohio	350	20.44	0.45
Colorado	250	45.98	0.54
Illinois	370	40.94	0.47
Texas	760	51.6	0.52
Nevada	420	77.93	0.51
Louisiana	310	73.52	0.43
Arkansas	400	46.83	0.46
Florida	550	29.47	0.42
Idaho	440	70.72	0.49
**Bottom 10 Avg**	***424***	***53.17***	***0.48***
Difference	94.0 (*p* = 0.20)	21.68 (*p* = 0.024)*	− 2.54 (*p* < 0.0001)*

^1^Reinert, M., Fritze, D., & Nguyen, T. (October 2022)."The State of Mental Health in America 2023"Mental Health America, Alexandria, VA

^2^Bureau of Health Workforce*,* Designated Health Professional Shortage Area Statistics.* Health Resources and Services Administration (HRSA), U.S. Department of Health and Human Services*

^3^NRI (2022). FY 2019 State Mental Health Agency Revenues and Expenditures. NRI’s 2020–2021 State MH Profile Highlights

^4^Centers for Medicare & Medicaid Services, Office of the Actuary, National Health Statistics Group (2022). National Health Expenditure Data: Health Expenditures by State of Residence

The average percent of the state population living in a mental health provider shortage area in the top ten states was 31.50% compared to 53.17% in the bottom ten states. This 21.68% percentage point difference was nearly, but not quite statistically significant (*p* = 0.054), indicating that states with higher expenditures and more comprehensive parity trend toward fewer residents living in shortage areas.

In terms of total health expenditure allocated to SMHAs, the average for the top ten states was 3.02%, while the average for the bottom ten states was 0.48%, resulting in a significant difference of 2.54 (*p* = 0.002).

The third hypothesis was that states with comprehensive parity would be more successful in the system quality indicators compared to those without comprehensive parity (see Table 4). The seven states with comprehensive parity had significantly greater mental health workforce availability (*p* = 0.01), and a significantly smaller proportion of their population lived in mental health provider shortage areas (*p* = 0.0089). Furthermore, these states allocated a significantly higher percentage of their total health expenditures to state mental health agencies (*p* = 0.0089).

**Table 4:** Mental health workforce availability, percent of state population living in a mental health provider shortage area, and percent of health expenditure spent on behavioral health for the top ten states and bottom ten states separated by parity status (comprehensive vs. not comprehensive)

State	Mental health workforce availability (population per 1 provider)^1^	% of state population living in mental health provider shortage areas^2^	% of total health expenditure spent on mental health by state agencies^3,4^
***Comprehensive parity***^***5***^			
Alaska	160	51.66	3.40
Vermont	200	0	3.38
New York	310	21.22	2.73
Pennsylvania	410	13.39	3.06
Oregon	170	39.03	3.05
Maine	190	19.32	2.42
Maryland	330	21.09	2.40
**Comprehensive Avg**	***252.86***	***23.67***	***2.92***
***Limited parity***^***5***^			
Iowa	570	57.57	2.93
Montana	300	53.10	2.70
Arizona	660	38.59	4.13
Kentucky	390	74.29	0.49
Ohio	350	20.44	0.45
Colorado	250	45.98	0.54
Illinois	370	40.94	0.47
Texas	760	51.6	0.52
Nevada	420	77.93	0.51
Louisiana	310	73.52	0.43
Arkansas	400	46.83	0.46
Florida	550	29.47	0.42
Idaho	440	70.72	0.49
**Limited Avg**	***443.85***	***52.38***	***1.12***
Difference	190.99 (*p* = 0.01)*	28.71 (*p* = 0.0089)*	− 1.80 (*p* = 0.0089)*

^1^Reinert, M., Fritze, D., & Nguyen, T. (October 2022). “The State of Mental Health in America 2023” Mental Health America, Alexandria, VA

^2^Bureau of Health Workforce*,* Designated Health Professional Shortage Area Statistics.* Health Resources and Services Administration (HRSA), U.S. Department of Health and Human Services*

^3^NRI (2022). FY 2019 State Mental Health Agency Revenues and Expenditures. NRI’s 2020–2021 State MH Profile Highlights

^4^Centers for Medicare & Medicaid Services, Office of the Actuary, National Health Statistics Group (2022). National Health Expenditure Data: Health Expenditures by State of Residence

^5^The Kennedy Forum. *State Parity Reports*. Parity Track

## Discussion

The analysis revealed a significant correlation between higher SMHA expenditures and more comprehensive parity, supporting the first hypothesis. States in the top ten for SMHA expenditure were significantly more likely to have comprehensive mental health parity (*p* = 0.0016). Only three of the top ten states (Arizona, Iowa, and Montana) had limited parity legislation, whereas all ten of the bottom ten states had limited parity legislation. This suggests that SMHA expenditures not only reflect the strength of a state’s mental health infrastructure but also indicate a commitment to prioritizing mental health from a policy perspective.

The authors also hypothesized that greater SMHA expenditures and more comprehensive parity would lead to better system quality indicators. This was partially supported by significant differences in one of the three system-level indicators: the percentage of total health expenditure spent on mental health by state agencies. Finally, the hypothesis that states with comprehensive parity would have improved workforce availability, fewer people living in a provider shortage area, and a proportionally greater investment in SMHAs was supported by the analysis, with significant differences identified in each of the system quality indicators.

### State rankings

The authors ranked states based on SMHA expenditures in fiscal year 2019. Given the most recent collection of this data was in 2019, which predates the COVID-19 pandemic and the associated rise in mental health needs, it is possible that current expenditures and, thus, rankings would differ. It is also important to note that this analysis was limited to state mental health agencies’ expenditures, excluding other public and private health expenditures for mental health services due to the available data. Some states may have low state agency expenditures but greater expenditures by other agencies, which this analysis could not capture. While these are important considerations, ranking states based on SMHA expenditures was the best option, given the currently available data.

### Parity evaluation

For parity implementation, as hypothesized, states with greater SMHA expenditures had more extensive parity implementation and better enforcement of parity. While the states with greater mental health expenditures by state agencies did have a smaller proportion of states with limited parity, three of the states (Arizona, Iowa, and Montana) still had limited parity legislation, suggesting improvements in parity are necessary even among some of the top ten states. Beyond this, six of the top ten states (Alaska, Arizona, Maine, Iowa, Montana, and Maryland) had no published parity enforcement, raising concerns about the lack of enforcement, even among states with comprehensive legislation. In the bottom ten states, all ten had limited parity legislation, and only one (Illinois) had evidence of enforcement of parity, suggesting necessary improvements in both the scope and enforcement of mental health parity.

### Systems of care

The systems of care measures—mental health workforce availability, percent of state population living in a mental health provider shortage area, and percent of total health expenditure spent on mental health by state agencies—each trended in the way the authors hypothesized, with one the three measures showing significant differences between the top and bottom ten states. The significant difference in the percentage of total health expenditure allocated to SMHAs between the top ten and bottom ten states suggests that states with low mental health expenditures are not spending less money on health in general, but rather, they are proportionately spending significantly less money on mental health. Thus, mental health is being discriminated against in the bottom ten states, and there is not a proportionate allocation of funds for mental health; this disparity likely reflects policy priorities and the undervaluation of mental health services.

To further explore how comprehensive mental health parity may be associated with system-level outcomes separately from SMHA expenditure, the authors also compared the three outcome measures by parity status for the top ten and bottom ten states. The seven states with comprehensive parity had significantly greater mental health workforce availability *(p* = 0.01), a lower percentage of the population living in mental health provider shortage areas (*p* = 0.0089), and allocated a significantly higher percentage of total health expenditures to SMHAs (*p* = 0.0089) compared to states without comprehensive parity. These findings further support the hypotheses that parity legislation is associated with not only higher investment in mental health services but also with improved access and resource distribution across state systems.

### Limitations

While authors acknowledge that some states including more categories of spending in their mental health expenditures is a limitation, this is not something they could account for in this paper, given the scope of these differences is unknown. While the authors originally planned to use Mental Health America rankings or the state report cards put out by the Kennedy Forum, the rankings for both included data on the system-level effects the authors were looking at. Therefore, the use of these rankings would have confounded the present analyses.

While the authors would have ideally utilized all data from the same year, given that many of these measures are tracked infrequently, they felt it was important to use the most up-to-date data for each measure.

The authors acknowledge that per capita state mental health agency expenditures are a function of many factors. However, accounting for potential confounding factors is beyond the scope of this paper as that data is not available.

Finally, though the authors used specific criteria for designating states as having limited legislation, it was still a subjective designation, and the degree to which the legislation was limited differed between states designated this way.

## Implications for Behavioral Health

One of the key implications of this report is the pressing need for more comprehensive and up-to-date state-level mental health data. SMHA expenditures have not been examined since 2019, and no current data is available on total state mental health expenditures. Given the profound shifts in the mental health landscape, particularly in the aftermath of the COVID-19 pandemic, regularly collecting this data has become crucial. Without timely and comprehensive data, policymakers cannot accurately assess the impact of current mental health policies or make informed decisions about future policy recommendations.

Another implication of this report is that parity does not go far enough. While parity legislation has made strides in addressing financial barriers to mental healthcare, it falls short in addressing other critical aspects of care, including quality of care, workforce availability, outcomes, and access beyond financial coverage.

The findings of this research underscore the importance of SMHA expenditures, which represent mental health infrastructure, in enhancing mental health parity implementation and improving system-level outcomes. States with higher per capita expenditures and more comprehensive parity legislation were generally more likely to have improved access to mental health services, as evidenced by a lower percentage of populations living in mental health provider shortage areas, greater workforce availability, and a higher proportion of total health expenditures allocated to mental health. These outcomes suggest that increased investment in mental health infrastructure, in terms of finances and policy, is essential to work toward a more equitable and accessible mental healthcare system.

This research highlights significant gaps in mental healthcare, particularly among the states with low expenditures, where there is less comprehensive parity legislation and enforcement, disproportionately low investments in mental health, and large percentages of the state population living in provider shortage areas. This suggests that policy priorities in these states are not sufficiently addressing mental health needs.

The authors hypothesized that states with greater expenditures by SMHAs would have more comprehensive parity implementation, which was supported by the analysis. Future research should prioritize the collection of more comprehensive system-level mental health data and should include the analysis of individual-level outcome measures to identify whether state-level parity implementation and better-performing behavioral healthcare systems translate to improved individual-level outcomes.

## Data Availability

This study did not generate any new data.

## Appendix Case Studies of Individual States

### Vermont Case Study

Vermont was ranked number two in the top ten states.

The Vermont Department of Mental Health falls under the Agency of Human Services and coordinates publicly funded mental health services; it coordinates both direct mental health services like the Vermont Psychiatric Care Hospital and oversees the Designated Agency (DA) for community-based services ^22^.

Vermont is one of the states with the most comprehensive parity laws. In Vermont law, parity is required for all “individual plans, small employer fully funded insured plans, large employer fully insured plans, and any state-administered insurance plans.” ^10^ Additionally, this coverage must include services for all behavioral conditions listed in the International Classification of Diseases (ICD) ^10^. Vermont also requires that behavioral healthcare and all other medical care have the same deductible, further ensuring no difference in financial requirements between mental healthcare and other medical healthcare.

Vermont is also the state with the second highest state mental health agency (SMHA) expenditures, both per capita at 411.12 and per capita for individuals below 138% of poverty at $3,051.19.^12^ Vermont has the second highest percent of total state health expenditures spent by SMHAs and is the only state reporting that none of the state population is living in a mental health provider shortage area. For each of the care access measures, Vermont performs better than average among the top ten states. For the problem measures of drug overdose mortality and suicide mortality, however, Vermont has rates higher than average among the top ten states.

The majority of funding, 97%, for the state mental health agency in Vermont comes from Federal and State Medicaid. This makes Vermont the state with the highest percent of funding from Medicaid ^11^. For the single-state agency for substance use disorders (SSA), 62% comes from Medicaid, 18% comes from state funds, 16% comes from SAMHSA block grants, and the remainder comes from other funding sources ^11^.

In terms of health insurance, 97% of Vermont residents have coverage, with 49% of those covered by private health insurance, 24% enrolled in Medicaid, and 21% enrolled in Medicare ^23^.

### Arizona Case Study

Arizona was ranked number five in the top ten states.

In Arizona, the Arizona Department of Health Services (ADHS) oversees public health initiatives including mental health services, but there is not a specific department of mental health. Arizona is divided into four Regional Behavioral Health Authorities (RBHAs), which are community-based organizations that administer behavioral health services to the county or counties under their jurisdiction. Each of the RBHAs also has its own crisis hotline. The state also provides behavioral health services on five Native American reservations ^24^.

Mental health parity in Arizona applies only to large employer fully insured plans and explicitly excludes substance use disorder (SUD) services [10]. Additionally, these plans are not required to cover mental health services and only need to follow parity regulations if they do offer this coverage ^10^. Because mental health parity regulations in Arizona are only required for large employer plans and exclude SUD services, they are considered limited in scope.

While parity in Arizona is not fully comprehensive, the passing of S.B. 1523, Jake’s Law, in 2020 has led to increased prioritization of mental health services in Arizona. Jake’s Law gives authorization to the Division of Insurance for parity enforcement, created the Children’s Behavioral Services Fund to provide services for uninsured or underinsured youth, increases follow-up services for patients at risk for suicide, established a Suicide Mortality Review Team, and created a Mental Health Parity Advisory Committee ^25^. The unanimous support for this law suggests the shifting of policy priorities in Arizona towards prioritizing mental health.

Arizona was among the top ten states both for overall per capita SMHA expenditures and SMHA expenditures per capita for individuals falling under 138% of poverty ^12^. Important to note is that Arizona is the only state where SMHA expenditures also include substance use disorder service expenditures [12]. Thus, it is possible that Arizona is only included in the top ten states for this reason. The majority of funding for the SMHA in Arizona comes from Medicaid, which provides 84% of SMHA funds. The remainder comes from state funds (11%), SAMHSA Block Grants (1%), and other funding sources (4%) ^11^.

In terms of health insurance in Arizona, 87.3% of the population was covered in 2018, with 44.5% covered by employer-sponsored health insurance, 15.6% covered by Medicare, 3.3% covered by Medicare and Medicaid, and 19.1% covered by Medicaid/CHIP ^26^.

Of the states in the top ten for State Mental Health Agency expenditures, Arizona had the worst outcomes for mental health workforce availability and had the lowest percentage of total health expenditure spent on mental health by state agencies. Among the top ten states, Arizona also had the highest percent of adults with any mental illness who did not receive treatment, the highest percent of adults with any mental illness reporting unmet need, and the highest percent of adults reporting fourteen or more mentally unhealthy days a month who could not see a doctor due to costs. Interestingly, this did not translate to the highest drug overdose mortality or suicide mortality.

### Colorado Case Study

Colorado was ranked number forty-three in the bottom ten states.

In Colorado, behavioral health services are coordinated by the Behavioral Health Administration (BHA) within the Department of Human Services (DHS). The Behavioral Health Administration was created in 2022 after the passing of HB22-1278 and will expand upon the programs previously run by the Office of Behavioral Health in the DHS ^27^. Because the BHA is fairly new, some of the expansions mandated by HB22-1278 are still in progress, including a behavioral health monitoring system and grievance system, a behavioral health safety net system, and regional behavioral health service organizations. This bill also appropriates funds to the Behavioral Health Administration ^27^. Thus, it is possible that this shift in policy priority towards mental health services will be reflected in greater SMHA expenditures in the coming years.

In current Colorado parity laws, behavioral health coverage is required to meet parity regulations for large employer fully insured plans, small employer fully insured plans, and individual plans. However, this parity requirement only applies to services for a select list of behavioral health conditions making it limited parity legislation ^10^.

Colorado is among the bottom ten both for states for overall per capita SMHA expenditures and per capita expenditures for the population under 138% of poverty. In Colorado, 73% of SMHA funding comes from state and federal Medicaid matching. The remainder comes from State Funds (23%), SAMHSA Block Grants (1%), and other funding sources (2%). For the SSA, 38% comes from state funds, 2% comes from Medicaid, 47% comes from SAMHSA block grants, and 13% comes from all other funding sources ^11^. Interestingly, while Colorado had low SMHA expenditures, it had a large workforce availability.

In terms of health insurance coverage in Colorado, 91.2% of the population had health insurance coverage in 2018. Of individuals with health coverage, 52.1% had employer-sponsored health insurance, 12.5% had Medicare, 2.5% had Medicare and Medicaid, and 16% had Medicaid/CHIP ^26^.

### Idaho Case Study

Idaho was ranked number fifty in the bottom ten states.

Idaho does not have a designated department for behavioral health services but rather has behavioral health services and programs embedded within the Idaho Department of Health and Welfare ^28^. Idaho currently has extremely limited parity. Not only does parity only apply to state government employees and their spouses and dependents, but it also only applies to seven behavioral health conditions. Thus, the majority of individuals in Idaho are not protected under parity at all ^10^.

There is a new Idaho Behavioral Health Plan (IBHP), which coordinates behavioral health services in Idaho and intends to expand behavioral health benefits for an estimated 425,000 Idaho residents (Health and Welfare). This plan is separate from mental health parity but will have a single care provider manage the behavioral health system in Idaho ^29^. This plan will manage behavioral health services for individuals on Medicaid, other insurance, and for individuals who do not have insurance. The date of implementation of this plan is not yet available, but it is possible that this plan will fill some of the gaps in behavioral health services that result from such limited parity legislation.

In Idaho, there are seven regions, each with its own regional behavioral health board. These boards advise the state Behavioral Health Planning Council on the behavioral health needs of their region and proposed programming for their region, identify gaps in services, and assist with managing service system improvements ^29^.

Idaho is the state with the lowest overall per capita SMHA expenditure and is among the bottom ten for SMHA expenditure per capita for individuals below 138% of poverty. It is important to note that in Idaho, Medicaid Revenues for Community Programs are not included in the SMHA expenditures. In Idaho, 72% of SMHA funding comes from state funds, 9% comes from Medicaid, 17% comes from other funding sources, and 1% comes from SAMHSA block grants ^11^. For the SSA, 60% of funding comes from State funds, 7% comes from Medicaid, 22% comes from SAMHSA block grants, and 11% comes from other funding sources ^11^.

In 2018, 86.8% of individuals in Idaho were covered under a health plan. 47.1% of individuals were covered under employer-sponsored plans, 14.6% were covered under Medicare, 3.4% were covered under Medicare and Medicaid, and 13.2% were covered under Medicaid/CHIP ^26^.

Of all fifty states, Idaho had the lowest per capita mental health expenditures by the State Mental Health Agency ^12^. Idaho also had the most restrictive parity. In Idaho parity legislation, only plans for state government employees and their spouses and children require mental health parity, and even this coverage only applies to a small list of serious mental disorders ^10^. Despite this, Idaho does not have the worst outcomes for any of the system measures, care access measures, or problem measures. This suggests that there are other factors affecting these measures and that parity may not have as significant an impact on outcomes as the authors initially hypothesized.
